# Identification and characterisation of NANOG+/ OCT-4^high^/SOX2+ doxorubicin-resistant stem-like cells from transformed trophoblastic cell lines

**DOI:** 10.18632/oncotarget.24151

**Published:** 2018-01-11

**Authors:** Reham M. Balahmar, David J. Boocock, Clare Coveney, Sankalita Ray, Jayakumar Vadakekolathu, Tarik Regad, Selman Ali, Shiva Sivasubramaniam

**Affiliations:** ^1^ School of Science and Technology, Nottingham Trent University, Clifton Lane, Nottingham NG11 8NS, United Kingdom; ^2^ The John van Geest Cancer Research Centre, Nottingham Trent University, Clifton Lane, Nottingham NG11 8NS, United Kingdom

**Keywords:** trophoblast, stem-like cells (SLCs), doxorubicin, embryonic stem cells (ESCs), chemoresistance

## Abstract

Treatment of gestational trophoblastic diseases (GTD) involves surgery, radiotherapy and chemotherapy. Although, these therapeutic approaches are highly successful, drug resistance and toxicity remain a concern for high risk patients. This Chemoresistance has also been observed in the presence of cancer stem cells that are thought to be responsible for cases of cancer recurrence. In this study, we report the presence of previously unknown populations of trophoblastic stem-like cells (SLCs) that are resistant to the chemotherapeutic drug doxorubicin. We demonstrate that these populations express the stem cell markers NANOG and Sox2 and higher levels of OCT-4 (NANOG+/OCT-4^high^/SOX2+). Although chemoresistant, we show that the invasive capacity of these trophoblastic SLCs is significantly inhibited by doxorubicin treatment. To better characterise these populations, we also identified cellular pathways that are involved in SLCs-chemoresistance to doxorubicin. In summary, we provide evidence of the presence of NANOG+/OCT-4+/SOX2+ trophoblastic SLCs that are capable to contribute to the susceptibility to GTD and that may be involved in Chemoresistance associated with drug resistance and recurrence in high risk GTDs’ patients. We propose that targeting these populations could be therapeutically exploited for clinical benefit.

## INTRODUCTION

Gestational trophoblastic diseases (GTD) are group of rare conditions that are associated with abnormal growth of trophoblast cells in the uterus. They can be caused by hydatiform moles and choriocarcinoma. Healthy trophoblastic cells are highly invasive toward the endometrium and are essential to the development of a rich uterine vasculature that is necessary for placenta formation [1–3]. However, in gestational trophoblastic disease the regulatory mechanisms fail, which result in tumours that are highly invasive, metastatic, and vascular [4]. Treatment of GTD involves surgery, radiotherapy and chemotherapy which can achieve 84-100% success depending on risk factors [5]. Unfortunately, some women die from the disease due to chemotherapy-associated toxicity and drug resistance [6]. Therefore, understanding the reasons associated with these limitations would improve the efficacy and help reduce drugs toxicity.

Chemotherapy is based on the ability of a drug to halt or destroy rapidly dividing cells. However, their efficacy is limited by two main issues: (i) their inability to differentiate between normal and highly dividing cells” such as hair follicles, intestinal epithelial and gonadal cells, and neoplastic cells [7]; (ii) the emergence of a self-renewing cell pool called “cancer stem cells” (CSC) within the tumour [8, 9]. The former is responsible for the unwanted side effects and the latter results in drug resistant tumours. Interestingly, in GTD, trophoblast cells of the human placenta can have similar behaviour to cancer stem cell-like cells [10] as they also demonstrate the ability to produce stem-like cells during first trimester invasion [11]. In fact, several similarities can be observed between tumour and trophoblast development [12, 13]. They both have a very high capacity to proliferate, differentiate, and invade surrounding tissues to establish a blood and nutrient supply by degrading the extra-cellular matrix (ECM) including changes in cell adhesion molecules, secretion of proteases, and growth factors [14, 15]. They also share other characteristics such immune response evasion, survival [12] and increased angiogenesis [16]. Based on this background, we hypothesised that transformed trophoblast cells contain populations of stem cell/cancer stem like cells that are resistant to chemotherapeutic agents. Furthermore, we reveal the existence of a population of trophoblast stem-like cells (SLCs) that express the stem cells markers OCT-4, SOX2, and NANOG and trophoblast transcription factor (CDX2) that are capable of self-renewal by generating spheroids.

We also investigated the effect of doxorubicin on these cells and we showed that within NANOG+/OCT-4+/^_^/SOX2+trophoblast SLCs only NANOG+/OCT-4^high^/SOX2+ populations are resistant to doxorubicin. We also show that the invasive capacity of these populations is significantly inhibited by doxorubicin treatment. Moreover, we investigated by proteomic analysis cellular pathways that are involved in trophoblast SLCs resistance to doxorubicin. Collectively, these studies demonstrate the existence of trophoblast SLCs that are chemoresistance and that may explain drug resistance observed in some cases of Gestational trophoblastic diseases.

## RESULTS

### Identification of stem-like cells (SLCs) from transformed trophoblastic cell lines

**Figure 1:**
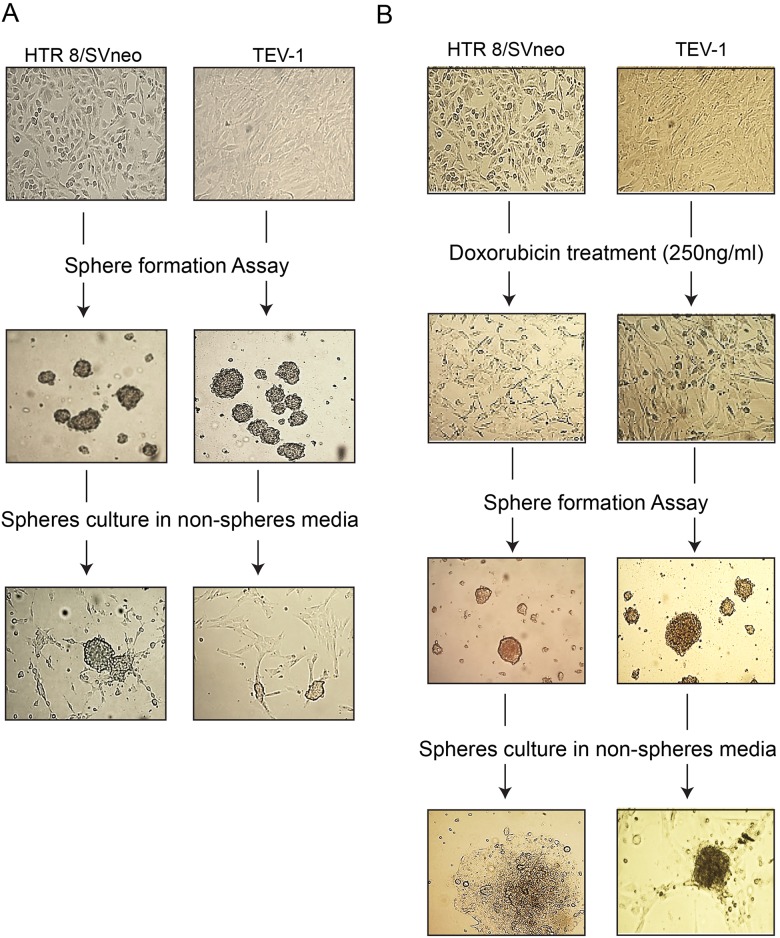
Generation of non-resistant and doxorubicin-resistant spheroids from transformed trophoblast cell lines The ability of trophoblast cells (HTR8/SVneo and TEV-1) to generate non-resistant spheroids are shown in Panels **(A)**. Row 1 = Parental cells without treatment; Row 2 = Spheroidal cells produced from non-adherent 3-D culture; Row 3 = the ability of spheroidal cells to re-grow onto normal adherent 2-D culture. Likewise, trophoblast cells (HTR8/SVneo, TEV-1) have the ability to generate doxorubicin-resistant spheroids shown in Panel **(B)**. Row 1 = Parental cells without treatment; Row 2=Parental cells treated with 250ng/ml of doxorubicin. Row3 = Spheroidal cells produced from non-adherent 3-D culture; Row 3 = the ability of spheroidal cells treated to re-grow onto normal adherent 2-D culture. Scale bar= 100μm.

**Figure 2:**
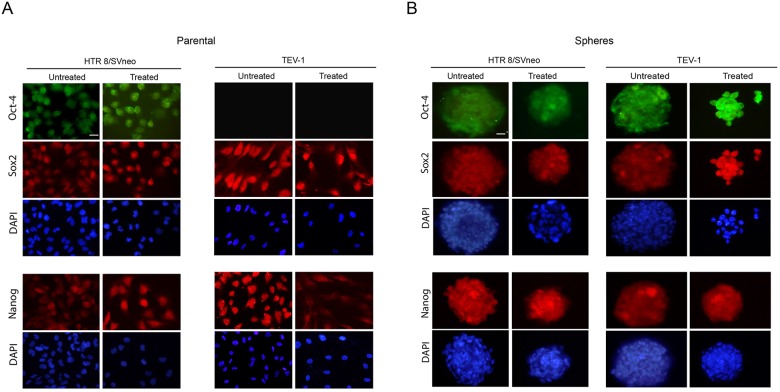
Expression of NANOG, OCT-4 and SOX2 in transformed trophoblast spheroids and parental cells **(A) (B)** Immunofluorescence micrographs of OCT-4, SOX2 and NANOG with DAPI staining in HTR8/Svneo and TEV-1 transformed parental trophoblast cell lines. (B) Immunofluorescence micrographs OCT-4, SOX2 and NANOG with DAPI staining in transformed trophoblast spheroids. Objective magnification 40X. Scale bar = 50μm.

As limited attempts have been made to identify SLCs from trophoblastic cells, we set out to investigate their presence in transformed first trimester trophoblastic cell lines (HTR8/SVneo and TEV-1). For this purpose, we used sphere formation assay that aims at testing the capacity of these cells to self-renew, a property associated with stemness. We observed that transformed trophoblastic cell lines are able to generate spheres (Figure 1A). These cells are also able to generate spheres following treatment with the chemotherapeutic drug doxorubicin (Figure 1B). For further characterisation, we investigated the expression of specific stem cell markers such as OCT-4, NANOG and SOX2 in parental and spheroid cells by immunofluorescence staining and using specific antibodies. Spheroids that were generated from trophoblastic cells showed positive staining for OCT-4, SOX2 and NANOG (Figure 2A and 2B). Although, HTR8/SVneo parental cells also expressed SOX2, NANOG and OCT-4, the expression of OCT-4 was not observed in TEV-1 parental cells (Figure 2A). These results demonstrate that trophoblastic cell lines can generate stem-like cells that are able to self-renew and express the stem cell markers OCT-4, NANOG and SOX2. These were confirmed both by QRTPCR (mRNA) and immunoblotting (protein) (Supplementary Figure 1A - 1D).

### Transformed trophoblastic stem-like cells (SLCs) are resistant to doxorubicin treatment

**Figure 3:**
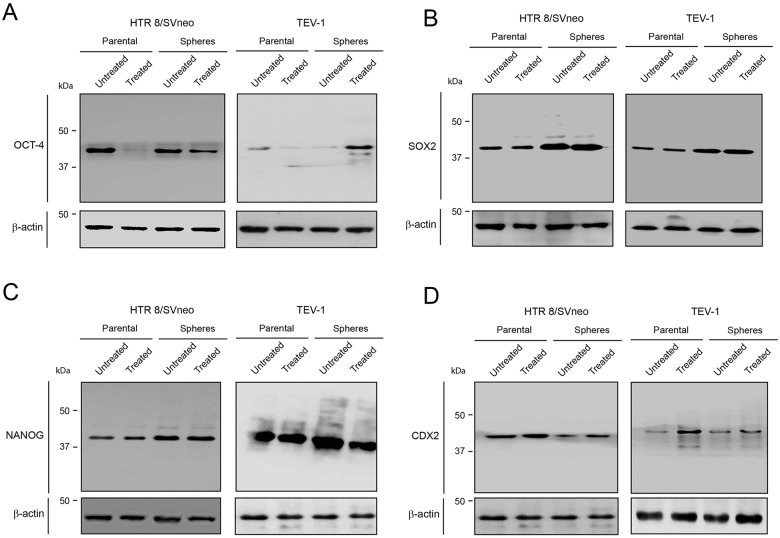
OCT-4, SOX2 and NANOG expression in untreated and treated transformed parental trophoblast cells and spheroids **(A) (B) (C) (D)** Immunoblots showing the expression of OCT-4, SOX2, NANOG, CDX2 and corresponding β-actin loading controls in HTR8/SVneo and TEV-1 untreated and treated transformed parental trophoblast cells and spheroids.

To investigate the effect of doxorubicin on trophoblastic stem-like cells, we treated transformed trophoblastic cells (parental) as well as spheroids that were originated from these cells with doxorubicin and we performed an immunofluorescence staining with OCT-4, NANOG and SOX2 antibodies. The expression of these markers was observed in both HTR8/SVneo and TEV-1 treated spheres. HTR8/SVneo parental cells expressed NANOG, SOX2 and OCT-4 (Figure 2A and 2B); however treated TEV-1 parental cells expressed only SOX2 and NANOG. Whole cell lysates from untreated and treated spheroids and parental cells were used for immunobloting with specific antibodies against OCT-4, NANOG and SOX2. Although we observed that the expression of OCT-4, NANOG and SOX2 was significantly increased in spheroids that were originated from trophoblastic cells, no increase of OCT-4 expression was noticed in TEV-1 trophoblastic cells (Figure 3). Conversely, an increase of OCT-4 expression was observed in doxorubicin-treated spheroids that were originated from TEV-1 (Figure 3A). This may be explained by the presence of OCT-4 SLCs a small subpopulation within TEV-1 SLCs that expresses NANOG and SOX2, and that are resistant to doxorubicin. Moreover, no significant difference was observed in NANOG and SOX2 expression between untreated and treated trophoblastic cells (Figure 3B and 3C). Although, OCT-4 expression was affected in treated-parental TEV-1, a decrease of OCT-4 expression was also observed in doxorubicin treated HTR8/SVneo (Figure 3A). Finally, there were no significant changes observed in protein expression of the trophoblastic marker CDX2 in HTR8/SVneo and TEV-1 cell lines. However, CDX2 expression was increased in doxorubicin-treated HTR8/SVneo spheroids compared to untreated spheroids (Figure 3D). These results demonstrate that NANOG+/OCT-4^high^/SOX2+ SLCs populations from trophoblastic cells are resistant to the chemotherapeutic effect of doxorubicin.

### Doxorubicin treatment inhibits invasion of spheres from transformed trophoblastic cell lines

**Figure 4:**
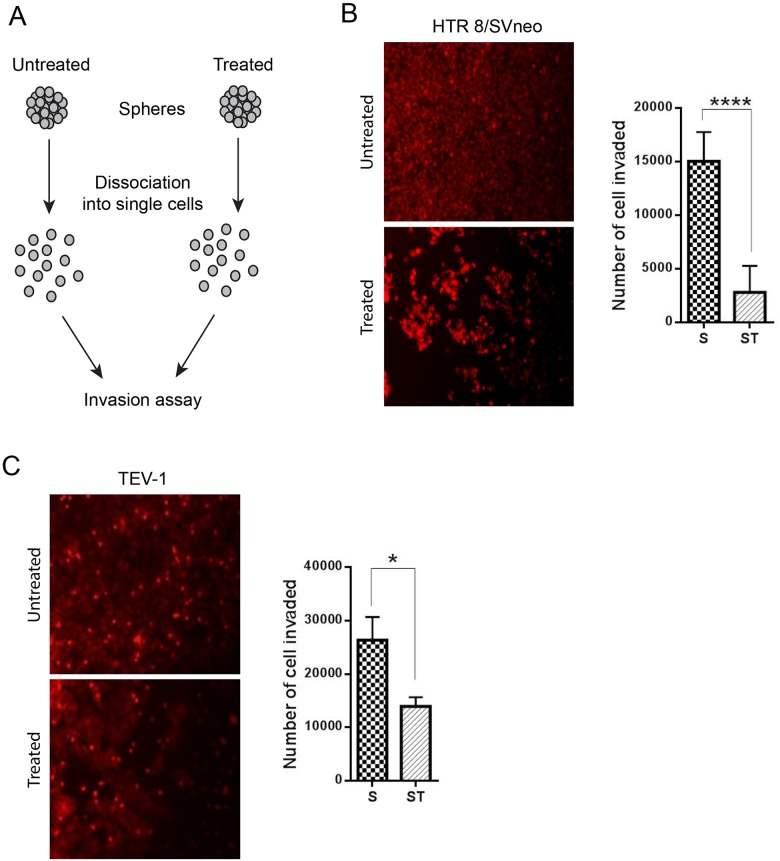
Comparative cell invasion of untreated and treated transformed trophoblast SLCs **(A)** Schematic representation of invasion assay. **(B) (C)** represent the number of HTR8/SVneo and TEV-1 cells invaded at 24 h. A t tests was carried out. Data represent the mean ±SD of three individual experiments, each performed in triplicate (^****^p<0.0001; ^*^p<0.05). Objective magnification 20X (Scale bar = 100 μm).

**Figure 5:**
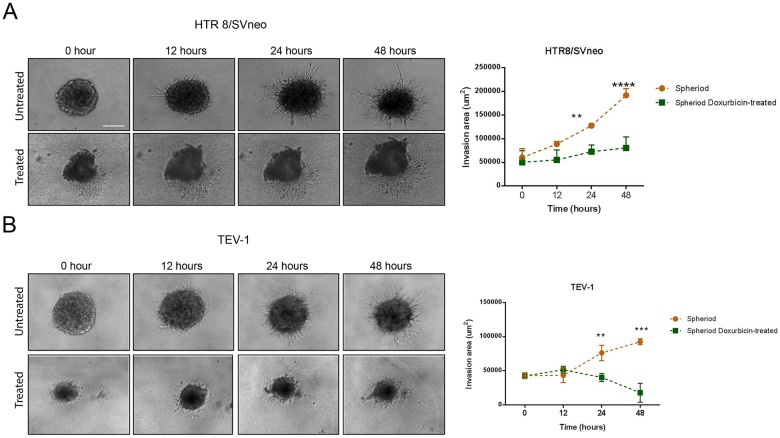
3-D Invasion of untreated and doxorubicin-treated spheroids **(A) (B)** Invasion pattern of untreated and treated HTR8/Svneo and TEV-1 spheroids and corresponding quantitative analysis of invasion areas. A two-way ANOVA followed by Sidak's for multiple comparisons was carried out. Data represent the mean ±SD of three individual experiments, each performed in quintuplicate (^****^p<0.0001; ^***^p<0.001; ^**^p<0.01). Objective magnification=10X.

To determine the effect of doxorubicin on the invasive capacity of spheres originated from transformed trophoblast cell lines, we used the BD BioCoat tumour invasion plates. This assay allows the study of the invading capacity of cells by comparing the number of cells invaded under different conditions. Prior to the invasion assay, the spheroids were dissociated into single cells using trypsin (Figure 4A). Doxorubicin-treated spheroids from the trophoblastic cells HTR8/SVneo and TEV-1 cells showed decreased invasive capacities when compared to untreated spheroids (Figure 4B and 4C). To further confirm these results, we used another cell invasion assay, the 3-D Spheroid BME cell invasion assays, to compare the invasive potential of untreated and doxorubicin-treated spheroidal cells. Image J software was used to analyse all confocal microscopy images and to measure changes in the invasion area at 12, 24 and 48 hrs. We observed a significant decrease of invasion of HTR8/SVneo doxorubicin-treated spheroids when compared to their untreated counterpart (Figure 5A). This decrease correlated with time. At 12 hrs, there was no significant difference in the invasion capacity between untreated and doxorubicin-treated spheroids. However, a significant increase was observed with untreated spheroidal cells at 24 and 48 hrs. This pattern of invasion was also noticed with TEV-1 untreated and treated spheroids (Figure 5B). These results demonstrate that the invasive property of the trophoblastic SLCs is inhibited by doxorubicin.

### Proteomic analyses of cellular pathways involved in trophoblastic SLCs response to doxorubicin treatment

To investigate potential cellular pathways that are involved in trophoblastic SLCs response to doxorubicin treatment, we performed proteomic analyses of untreated and treated HTR8/SVneo and TEV-1 spheroids. Proteins with significant differential expression were selected for further analysis using Metacore software (Thomson Reuters, Tables 1 and 2). In the HTR8/SVneo model, we identified several cellular pathways that were activated. These pathways are involved in cytoskeleton remodelling, cell adhesion and migration, cell cycle initiation, apoptosis and survival, and immune response (Supplementary Table 1). Among the highly expressed proteins MyHC (Myosin Heavy Chain) was found to be associated with most of these pathways and this suggests a potential key role in trophoblastic SLCs response to doxorubicin. Conversely, pathways that are involved in glycolysis and gluconeogenesis, spindle assembly and transport were negatively regulated (Supplementary Table 2). In the TEV-1 model, we identified 1 main activated pathway that is associated with cytoskeleton remodelling mediated by PKA (Protein Kinase A) (Supplementary Table 3). The LIM and SH3 domain protein 1 (LASP1) appears to play a key role in this process. This was confirmed by immunoblotting (Supplementary Figure 2). On the other hand, several cellular pathways are downregulated and are involved in immune response mediated by the Major Histocompatibility Class I (MHC class I), ubiquitin pathway, CFTR folding and maturation pathway, and apoptosis and survival (Supplementary Table 4). The dowregulation of these pathways appears to be due to decreased expression of Calreticulin, PDIA3 (Protein Disulfide Isomerase Family A Member 3), HSP70 (Heat Shock Protein Family A) and GRP78 (Glucose-Regulated Protein, 78kDa). Although, treated HTR8/SVneo and TEV-1 spheroids appear to respond differently to doxorubicin treatment, the cytoskeleton remodelling pathway appears to be one of the key upregulated cellular process as it is found upregulated in both HTR8/SVneo and TEV-1 spheroid models.

**Table 1:** Protein expression changes following treatment in HTR8/SVneo spheroids Quantitation is by SWATH-MS and data analysis in the SCIEX OneOmics cloud processing platform (n=4 biological replicates, OneOmics confidence over 60%

	Protein		Absolute Fold Change	OneOmics Confidence %
Up in HTR Spheres Treated	RPN1_HUMAN	Dolichyl-diphosphooligosaccharide--protein glycosyltransferase subunit 1	5.28	68.3
	CALX_HUMAN	Calnexin	4.40	70.9
	BASI_HUMAN	Basigin	3.55	70.7
	STOM_HUMAN	Erythrocyte band 7 integral membrane protein	2.78	80.6
	VTNC_HUMAN	Vitronectin	2.35	72.6
	COX5A_HUMAN	Cytochrome c oxidase subunit 5A, mitochondrial	2.27	61.7
	PHB_HUMAN	Prohibitin	1.68	80.4
	NASP_HUMAN	Nuclear autoantigenic sperm protein	1.68	65.5
	NHRF1_HUMAN	Na(+)/H(+) exchange regulatory cofactor NHE-RF1	1.63	73.7
	LMNB2_HUMAN	Lamin-B2	1.53	60.5
	MYH9_HUMAN	Myosin-9	1.51	85.7
	PHB2_HUMAN	Prohibitin-2	1.51	65.7
	CLCA_HUMAN	Clathrin light chain A	1.48	65.6
	K2C8_HUMAN	Keratin, type II cytoskeletal 8	1.46	66.0
	GLU2B_HUMAN	Glucosidase 2 subunit beta	1.44	79.3
Down in HTR Spheres Treated	RAN_HUMAN	GTP-binding nuclear protein Ran	−1.19	64.9
	RLA0_HUMAN	60S acidic ribosomal protein P0	−1.46	61.1
	PGK1_HUMAN	Phosphoglycerate kinase 1	−1.47	84.2
	ENOG_HUMAN	Gamma-enolase	−1.48	79.7
	G3P_HUMAN	Glyceraldehyde-3-phosphate dehydrogenase	−1.55	63.2
	1433T_HUMAN	14-3-3 protein theta	−1.60	68.7
	G6PI_HUMAN	Glucose-6-phosphate isomerase	−1.63	69.6
	PPIB_HUMAN	Peptidyl-prolyl cis-trans isomerase B	−1.65	70.2
	ACADV_HUMAN	Very long-chain specific acyl-CoA dehydrogenase, mitochondrial	−1.69	69.9
	GNPI1_HUMAN	Glucosamine-6-phosphate isomerase 1	−1.81	72.8

**Table 2:** Protein expression changes following treatment in TEV-1 spheroids Quantitation is by SWATH-MS and data analysis in the SCIEX OneOmics cloud processing platform (n=4 biological replicates, OneOmics

	Protein		Absolute Fold Change	OneOmics Confidence %
Up	COTL1_HUMAN	Coactosin-like protein	2.123830573	0.669641101
	RL21_HUMAN	60S ribosomal protein L21	1.918845638	0.636431099
	EF1G_HUMAN	Elongation factor 1-gamma	1.607650899	0.739079333
	LASP1_HUMAN	LIM and SH3 domain protein 1	1.512337143	0.605927912
	RSSA_HUMAN	40S ribosomal protein SA	1.453157634	0.676316894
Down	TPIS_HUMAN	Triosephosphate isomerase	−1.499305054	0.608419546
	PDIA3_HUMAN	Protein disulfide-isomerase A3	−1.781548127	0.746982791
	ML12A_HUMAN	Myosin regulatory light chain 12A	−1.992053636	0.684952806
	PDIA1_HUMAN	Protein disulfide-isomerase	−2.023966219	0.701529248
	GRP78_HUMAN	78 kDa glucose-regulated protein	−2.756855044	0.898932262
	CALR_HUMAN	Calreticulin	−2.993779141	0.62324947
	VTNC_HUMAN	Vitronectin	−3.815950175	0.671235282

## DISCUSSION

Chemotherapy is based on the ability of a drug to halt or destroy rapidly dividing cells. However, their efficacy is limited by (i) their inability to differentiate between normal and highly dividing cells [6]; (ii) the emergence of a self-renewing cell pool called “cancer stem cells” (CSC) within the tumour [7, 17, 18]. In GTDs, similar concerns are associated with chemotherapy-associated toxicity and drug resistance in high risk patients. These therapeutic limitations may also be due to the existence of tumorigenic trophoblastic SLCs that confers resistance to chemotherapy. Therefore, identifying and characterising these populations is essential to help improve the efficacy of chemotherapy for patients at high risk of GTDs. Historically, two different types of trophoblast cells have been used as *in vitro* models (a) transformed first trimester trophoblast cell lines [19–21]; and (b) choriocarcinoma which were originated from trophoblast tumours [22–24]. As we aimed to understand whether the early trophoblast cells (per se) are capable of producing SLC's contributing to the susceptibility of GTD, the cell lines derived from choriocarcinoma (such as Jar, JEG-3, or BeWo) are not suitable for this study. In contrast the cell lines HTR8/SVneo and TEV-1 were transformed using viral vectors and proved to show the physiological behaviours of early first trimester trophoblast cells [19–21].

In this study, we have identified populations of NANOG+/OCT-4+/^_^/SOX2+ trophoblastic SLCs that are able to self-renew and to generate spheroids. Interestingly, only populations that are NANOG+/OCT-4^high^/SOX2+ are resistant to doxorubicin treatment. This finding pinpoints to the fact that only a subpopulation of transformed trophoblastic SLCs (NANOG+/OCT-4^high^/SOX2+) resist to doxorubicin treatment and implies that this subpopulation might be responsible for at least cases of GTDs’ chemoresistance.

Cancer stem cells are also associated with invasion and metastasis following chemotherapy [25–29]. In this regard, we have investigated the invasion capacity of identified chemoresistant trophoblastic SLCs and in response to doxorubicin. Although, the invasion of transformed trophoblastic SLCs was significantly decreased, these results suggest that following chemotherapy, these populations of SLCs may re-initiate the invasive process that could lead to more aggressive forms of GTDs. In fact, Gestational trophoblastic disease (GTD) comprises a spectrum of disorders from the pre-malignant conditions of complete and partial hydatidiform moles to the malignant invasive mole (choriocarcinoma) and in very rare cased placental site trophoblastic tumour/epithelioid trophoblastic tumour (PSTT/ETT) [29]. These populations may contribute to these transitions by adopting aggressive phenotypes that are associated with cancer stem cells.

Furthermore, proteomic analyses of transformed trophoblastic SLCs led to the identification of several cellular pathways that may play an important role in their response to doxorubicin. Cytoskeleton remodelling pathways were identified as highly upregulated in treated SLCs spheroids. These pathways are involved in cell adhesion, migration and invasion. Although this is not surprising, the results confirm our observations with regard to SLCs capacity to generate spheroids which involves cell-cell adhesions and the anti-invasive effect in response to doxorubicin. Other cellular pathways such as spindle assembly and glucose metabolism were negatively regulated. Spindle assembly is an important step in cell division and the downregulation of this pathway could be explained by the known effect of doxorubicin on the cell cycle of dividing cells. Increased glucose metabolism has been associated with embryonic stem cells and is considered as one of the hallmarks of cancer stem cell metabolism [30, 31]. The downregulation of this pathway could be explained by the elimination of non-resistant trophoblastic SLCs (NANOG+/OCT-4+/^_^/SOX2+) or the slow-down of glucose metabolism in doxorubicin-resistant SLCs (NANOG+/OCT-4^high^/SOX2+). Finally, increased levels of expression of OCT-4 correlated with chemoresistance in several cancers [32], which suggests potential involvement of OCT-4 in the chemoresistance that is observed in our study. Taken together, these observations highlight the presence in transformed trophoblast of a population of SLCs (NANOG+/OCT-4^high^/SOX2+) that are resistant to doxorubicin treatment and provide further insights on the role of trophoblastic SLCs in GTDs associated-chemoresistance.

## MATERIALS AND METHODS

### Antibodies

For this study, we used anti-OCT4 (1:200 for IF, 1:500 for WB, ab18976 Abcam), anti- SOX2 (1:500 for IF, 1:1000 for WB, ab97959 Abcam), anti-NANOG (1:50 for IF, 1:500 for WB, OAAB11202, Aviva Systems Biology), anti-CDX2 (1:100 for IF, ab15258 Abcam; 1:500 for WB, ab88129 Abcam), anti-beta actin (1:1000 for WB, ab8227 Abcam) and Anti-LASP1 (1:1000 for WB, ab156872 Abcam).

### Cell lines and growth conditions

Two cell lines were used in this study. Two include HTR8/SVneo (kindly provided by Dr Charles Graham, University of Kingston, Canada) and TEV-1 (obtained from Dr Mei Choi Choey, University of Hong Kong, China). These cell lines were cultured in RPMI-1640 medium. The media were supplemented with 10% (v/v) FBS (foetal bovine serum) (Lonza), 1% (w/v) L-glutamine (Lonza) and 1% (v/v) penicillin/streptomycin (Lonza). Cells were grown in T25 flasks, incubated at 37°C in 5% (v/v) CO_2_ under humid conditions.

### Generation of non-resistant and drug-resistant (Doxorubicin) spheroids

The Spheroids were produced under two different conditions for the production of non-resistant spheroids cells were grown under normal cell culture conditions for 72 hrs. After 72 hrs, cells were trypsinised and 5 × 10^6^ cells were seeded in an ultra-low attachment (non-adherent) T75 flask in 20 mL of serum-free media. This media contained (1% w/v L-glutamine and 1% v/v penicillin/streptomycin) supplemented with growth factors, insulin and 0.4% BSA. The spheroid generation was monitored and they were separated from the single cells by subjecting them to gravity separation. Later these spheroids were harvested by centrifugation and allowed to grow for 72 hrs. The same methodology was followed for producing drug resistant spheroids except for the treatment of cells with 250 ng/mL of doxorubicin before performing aforementioned steps. The expression of various markers was later studied in both non-resistant and resistant spheroids using immunofluorescence, QRTPCR and immunoblotting.

### Immunofluorescence staining

Spheroids were collected from the non-adherent flask and transferred into 15 mL universal tubes by centrifuging at 500 x *g* for 10 mins, and then washed with PBS. The collected spheroids were fixed in 4% formaldehyde for 30 min at room temperature and washed three times with PBS. Nonspecific reactivity was blocked by incubating the cells in blocking solution (1% BSA in PBS Tween20) for 1 h. Subsequently the cells were subjected to primary antibody treatment for overnight at 4°C followed by the treatment with secondary antibody. After washing with PBS, cells were then transferred onto slides and mounted with two drops of VECTASHIELD^®^ HardSet™ mounting medium and the boundaries were secured by means of a hydrophobic image barrier pen (Vector Laboratories, Inc). Spheroid images were captured under fluorescent microscope using an Olympus camera. Parental adherent cells were grown onto sterile glass cover slips GG-18-PLL (neuVitro), poly-l-lysine coated cover-slips under the respective growth conditions and was used as control.

### RNA extractions and quantitative real-time PCR (qRT-PCR)

QRT-PCR was carried out to determine the mRNA expression level. RNeasy^®^ Plus Mini-kit (Qiagen, Inc) was used to extract the total of RNA from cell lines according to manufacturer's instructions. The RNA integrity and purification was determined by denaturing 1.5% agarose gel electrophoresis. The purity and concentration of RNA was determined by using Nanodrop 2000 spectrophotometer (Thermo Scientific) at a wavelength of 260 nm. Complementary DNA (cDNA) was then synthesized from RNA extracted using SuperScript™ II Reverse Transcriptase kit (Invitrogen^®^) according to manufacturer's instructions. The primers were designed using the Primer 3^®^ Input version 4 software. Details of primers (supplied by MWG Eurofin, Germany). The sequences of forward and reverse primers were: **OCT-4** (NCBI accession no. NM_001285986.1; 5’ AATTTGTTCCTGCAGTGCCC 3’ and 5’ CTCTCGTTGTGCATAGTCGC 3’), **SOX2** (NCBI accession no. NM_003106.3; 5’ CGGAAAACCAAGACGCTCAT 3’ and 5’TTCATGTGCGCGTAACTGTC 3’), **NANOG** (NCBI accession no. NM_001297698.1; 5’CCATCCTGCAAATGTCTTCTG 3’ and 5’ CTTTGGGACTGGTGGAAGAAT 3’), **CDX2** (NCBI accession no. NM_001265.4; 5’ GGGAGGACTGGAATGGCTAC 3’ and 5’ CCCAGAAGCGCAGGAAGG 3’). The qRT-PCR was performed using Rotor-Gene 6000 real time PCR cycler (Qiagen). The mRNA was normalised against averaged expression of the house-keeping genes [hypoxanthine phosphoribosyltransferase-1 (HPRT1) and TATA Box Binding Protein (TBP1)] in the same samples and 2-ΔΔCt was calculated.

### Immunoblotting

Harvested cells (both parental and spheroidal) were lysed directly in lysis buffer to collect whole cell extracts. Cells were washed twice with PBS then transferred into 20 mL universal tubes and centrifuged at 27,000 x g for 15 mins at 4°C. The supernatant was discarded and the pellet was washed with PBS. To the cells, 300 μL RIPA buffer with 0.2 mL protease inhibitor cocktail (Roche) 0.2% (v/v) and 1mM Na_3_VO_4_ (Sigma Aldrich) were added and the resulting cell lysate was boiled for 5 min at 95°C followed by storing the lysate −20°C and used whenever needed. Protein samples (30 μg) were separated using (SDS/PAGE; 10% (w/v) polyacrylamide gel) using a Bio-Rad Mini-Protean III system which were then transferred to nitrocellulose membranes using a Bio-Rad Trans-Blot system for 1 h at 100 volts in (25 mM Tris, 192 mM glycine, and 20% MeOH). Following transfer, the membranes were washed with PBS and blocked for 1 h at room temperature in blocking buffer (3% w/v BSA in 1X TBS-Tween20). The membranes were incubated overnight at 4°C with respective primary antibodies and incubated with the respective secondary antibody for 1-2 h. The ultra chemiluminescence detection system (Cheshire Sciences Ltd) was used to visualize the bands, and they were quantified by densitometry using Advanced Image Data Analysis Software (Fuji; version 3.52). The expression levels of the proteins of interest were normalised against the house keeping protein β-actin.

### Invasion assay

To compare the invasion/migration capacities of spheroidal cells with their parental counterparts, a 2-D invasion assay was carried out using the BD Falcon^™^ BioCoat tumour invasion systems (BD Falcon) with fluoroBlock^™^ 96 well insert plate according to manufacturers guidelines. This was also compared with migration of cells through uncoated BD Falcon^™^ FluoroBlok^™^ 96 well insert plates. The assay used had following steps Rehydration by filling 75 μL of warm media and allowed to hehydrated it for 2 hrs at 37°C at 5% v/v CO_2_. Pre-staining the cells by incubating with 10 μM Cell Trace^™^ CFSE red dye (Molecular Probes^®^) for 45 min. After incubation the cells were centrifuged and re-suspended in serum free media. The cell suspension was also added to the apical chambers BD falcon fluoroBlock^™^ 96 well migration plate insert. The bottom of the chambers was filled with growth media containing 5% v/v FBS. For Doxorubicin resistant cells, the apical as well as the bottom chambers were treated with 250 ng/mL of Doxorubicin. After incubating the plates for 24 hrs at 37°C and 5% CO_2_ the membranes were removed from the inserts and mounted on slides. The fluorescence of invaded cells was then read at a wavelength of 494/517 nm (Ex/ Em). The number of cells invaded from the tumour invasion plate together with percentage invasion (see below for equation) was analysed using Image J software.

$$Invasion\;\%=\frac{Number\;of\;cells\;invaded}{Number\;of\;cells\;migrated}\times 100$$

### Cultrex^®^ 96 well 3-D spheroid Basement Membrane Extract (BME) cell invasion assay

It should be noted that monolayer cell invasion systems are commonly used to evaluate invasion of single cells. Therefore, this method is not sufficient to study the invasion potential of a spheroid. Although the spheroids were artificially separated into single cells to suit the 2-D invasion assay, it is not entirely appropriate to compare the invasive behaviour of untreated and doxorubicin-treated spheroids a further experiment 3-D invasion assay was carried out. The assay was carried out according to the manufacturer's guidelines (Cultrex^®^ 3-D spheroid cell invasion assay Amsbio). The plate was then placed under a confocal microscope for imaging at 4 hrs intervals for 48 h. Image J software was used to analyse all confocal images, to measure changes in the invasion area at 12, 24 and 48 h.

### Statistical analyses

All data are presented as means ± SD. Statistical significance was determined using a one-way ANOVA followed by Tukey's and two-way ANOVA followed by Sidak's test for multiple comparisons and t tests p < 0.05 was considered statistically significant. (GraphPad Prism, version 6, California, USA).

### Mass spectrometry analysis

Cell lysates containing 50 μg protein were reduced and alkylated (1 μL 0.5 M DTT, 56°C for 20 min; 2.7 μL 0.55 M iodoacetamide, room temperature 15 min in the dark), evaporated to dryness in a vacuum concentrator (Eppendorf, UK) and resuspended in 100 μL 50 mM tri-ethyl ammonium bicarbonate (TEAB). Trypsin (2 μg in 2 μL of 1 mM HCl), was added in and incubated in a thermomixer overnight at 37°C. Samples were then again evaporated to dryness and resuspended in 5% (v/v) acetonitrile/0.1% (v/v) formic acid (20 μL) and transferred to a HPLC vial for MS analysis. For IDA (Information Dependent Analysis) to generate a spectral library, 8 μL of pooled sample in triplicate were injected by autosampler (Eksigent nanoLC 425 LC system) in microflow at 5 μL/min directly onto a YMC Triart-C_18_ column (15 cm, 3 μm, 300 μm i.d.) using gradient elution (2-40% Mobile phase B, followed by wash at 80% B and re-equilibration) over 87 min. For SWATH/DIA (Data Independent Analysis), 3 μL was injected on the same gradient elution profile over 57 min. Mobile phases consisted of A: 0.1% formic acid; B: acetonitrile containing 0.1% (v/v) formic acid. IDA analysis was carried out in positive ion mode with a 250 ms survery scan, *m/z* range 400-1250; Top 30 peaks selected for fragmentation, accumulation time 50 ms per experiment, cycle time 1.8 s. SWATH analysis used 100 variable windows, 25 ms per window, 100-1500 *m/z* using the SCIEX Duospray source with a 50 μm electrode at 5500v. Spectral libraries were constructed from three IDA runs of pooled samples spiked with HRM-kit retention time peptides (iRT, Biognosys AG, Switzerland), searched using ProteinPilot 5.0 (SCIEX, UK) against the Swissprot human database (Jan 2016). The library was aligned against the iRT peptides and the SWATH data extracted against this library using SCIEX OneOmics (SWATH Proteomics Cloud Toolkit) with the parameters 6 peptides per proteins, 6 transitions per peptide, XIC width 75ppm, 5 min extraction window. Analysis of 4 biological replicates per group was carried out in Protein Expression Workflow within OneOmics to identify significantly differentially expressed proteins, excluding proteins with only single peptides.

## SUPPLEMENTARY MATERIALS FIGURES AND TABLES











## Abbreviations

CFTR: Cystic fibrosis transmembrane conductance regulator
CSC: Cancer Stem Cells
GRP: Glucose-Regulated Protein
HSP: Heat Shock Protein Family
GTD: Gestational Trophoblast Disease
LASP1: LIM and SH3 domain protein 1
MyHC: Myosin Heavy Chain
PDIA3: Protein Disulfide Isomerase Family A Member 3
PKA: Protein Kinase A
SLC: Stem-Like- Cells

## References

[1] Aplin JD (1991). Implantation, trophoblast differentiation and haemochorial placentation: mechanistic evidence *in vivo* and *in vitro*. J Cell Sci.

[2] Zhu JY, Pang ZJ, Yu YH (2012). Regulation of trophoblast invasion: the role of matrix metalloproteinases. Rev Obstet Gynecol.

[3] Burton GJ, Fowden AL (2015). The placenta: a multifaceted, transient organ. Philos Trans R Soc Lond B Biol Sci.

[4] Louwen F, Muschol-Steinmetz C, Reinhard J, Reitter A, Yuan J (2012). A lesson for cancer research: placental microarray gene analysis in preeclampsia. Oncotarget.

[5] Seckl MJ, Sebire NJ, Berkowitz RJ (2010). Gestational trophoblastic disease. Lancet.

[6] Powles T, Savage PM, Stebbing J, Short D, Young A, Bower M, Pappin C, Schmid P, Seckl MJ (2007). A comparison of patients with relapsed and chemo-refractory gestational trophoblastic neoplasia. Br J Cancer.

[7] Saini RK, Chouhan R, Bagri LP, Bajpai AK (2012). Strategies of targeting tumors and cancers. J Cancer Res.

[8] Yoo YD, Han DH, Jang JM, Zakrzewska A, Kim SY, Choi CY, Lee YJ, Kwon YT (2013). Molecular characteristics of cancer stem-like cells derived from human breast cancer cells. Anticancer Res.

[9] Sainz B, Carron E, Vallespinós M, Machado HL (2016). Cancer stem cells and macrophages: implications in tumor biology and therapeutic strategies. Mediators Inflamm.

[10] Red-Horse K, Zhou Y, Genbacev O, Prakobphol A, Foulk R, McMaster M, Fisher SJ (2004). Trophoblast differentiation during embryo implantation and formation of the maternal-fetal interface. J Clin Invest.

[11] Burrows TD, King A, Loke YW (1996). Trophoblast migration during human placental implantation. Hum Reprod.

[12] Ferretti C, Bruni L, Dangles-Marie V, Pecking AP, Bellet D (2007). Molecular circuits shared by placental and cancer cells, and their implications in the proliferative, invasive and migratory capacities of trophoblasts. Hum Reprod.

[13] Holtan SG, Creedon DJ, Haluska P, Markovic SN (2009). Cancer and pregnancy: parallels in growth, invasion, and immune modulation and implications for cancer therapeutic agents. Mayo Clin Proc.

[14] Bulmer JN, Morrison L, Johnson PM (1988). Expression of the proliferation markers Ki67 and transferrin receptor by human trophoblast populations. J Reprod Immunol.

[15] Soundararajan R, Rao AJ (2004). Trophoblast ‘pseudo-tumorigenesis’: significance and contributory factors. Reprod Biol Endocrinol.

[16] Yang J, Weinberg RA (2008). Epithelial-mesenchymal transition: at the crossroads of development and tumor metastasis. Dev Cell.

[17] Tang DG (2012). Understanding cancer stem cell heterogeneity and plasticity. Cell Res.

[18] Al-Hajj M, Clarke MF (2004). Self-renewal and solid tumor stem cells. Oncogene.

[19] Graham CH, Hawley TS, Hawley RG, MacDougall JR, Kerbel RS, Khoo N, Lala PK (1993). Establishment and characterization of first trimester human trophoblast cells with extended lifespan. Exp Cell Res.

[20] Weber M, Knoefler I, Schleussner E, Markert UR, Fitzgerald JS (2013). HTR8/SVneo cells display trophoblast progenitor cell-like characteristics indicative of self-renewal, repopulation activity, and expression of “stemness-” associated transcription factors. Biomed Res Int.

[21] Feng HC, Choy MY, Deng W, Wong HL, Lau WM, Cheung AN, Ngan HY, Tsao SW (2005). Establishment and characterization of a human first-trimester extravillous trophoblast cell line (TEV-1). J Soc Gynecol Investig.

[22] Liu F, Soares MJ, Audus KL (1997). Permeability properties of monolayers of the human trophoblast cell line bewo. Am J Physiol.

[23] Orendi K, Kivity V, Sammar M, Grimpel Y, Gonen R, Meiri H, Lubzens E, Huppertz B (2011). Placental and trophoblastic *in vitro* models to study preventive and therapeutic agents for preeclampsia. Placenta.

[24] Pattillo RA, Gey GO (1968). The establishment of a cell line of human hormone-synthesizing trophoblastic cells *in vitro*. Cancer Res.

[25] Shiozawa Y, Nie B, Pienta KJ, Morgan TM, Taichman RS (2013). Cancer stem cells and their role in metastasis. Pharmacol Ther.

[26] Frank NY, Schatton T, Frank MH (2010). The therapeutic promise of the cancer stem cell concept. J Clin Invest.

[27] Shuang L, Qin L (2014). Cancer stem cells and tumor metastasis. Int J Oncol.

[28] Regad T (2017). Tissue-specific cancer stem cells: reality or a mirage?. Transl Med Rep.

[29] Seckl MJ, Sebire NJ, Fisher RA, Golfier F, Massuger L, C; Sessa (2013). ESMO Guidelines Working Group. Gestational trophoblastic disease: ESMO clinical practice guidelines for diagnosis, treatment and follow-up. Ann Oncol.

[30] Huang R, Rofstad EK (2017). Cancer stem cells (CSCs), cervical CSCs and targeted therapies. Oncotarget.

[31] Sancho P, Barneda D, Heeschen C (2016). Hallmarks of cancer stem cell metabolism. Br J Cancer.

[32] Villodre ES, Kipper FC, Pereira MB, Lenz G (2016). Roles of OCT4 in tumorigenesis, cancer therapy resistance and prognosis. Cancer Treat Rev.

