# Supplementary material for: Identification and characterisation of NANOG+/ OCT-4high/SOX2+ doxorubicin-resistant stem-like cells from transformed trophoblastic cell lines
Source: Oncotarget. 2018 Jan 11;9(6):7054–65. doi: 10.18632/oncotarget.24151 (PMC5805535; doi:10.18632/oncotarget.24151)
Supplement: Supplementary file 1 [file oncotarget-09-7054-s001.pdf]

Identification and characterisation of NANOG+/ OCT-4^{high}/ SOX2+ doxorubicin-resistant stem-like cells from transformed trophoblastic cell lines

SUPPLEMENTARY MATERIALS

Supplementary Table 1: HTR8/SVneo Spheres untreated vs treated up-regulated pathways.

See Supplementary File 1

Supplementary Table 2: HTR8/SVneo Spheres untreated vs treated down-regulated pathways.

See Supplementary File 2

Supplementary Table 3: TEV-1 Spheres untreated vs treated up-regulated pathways.

See Supplementary File 3

Supplementary Table 4: TEV-1 Spheres untreated vs treated down-regulated pathways.

See Supplementary File 4

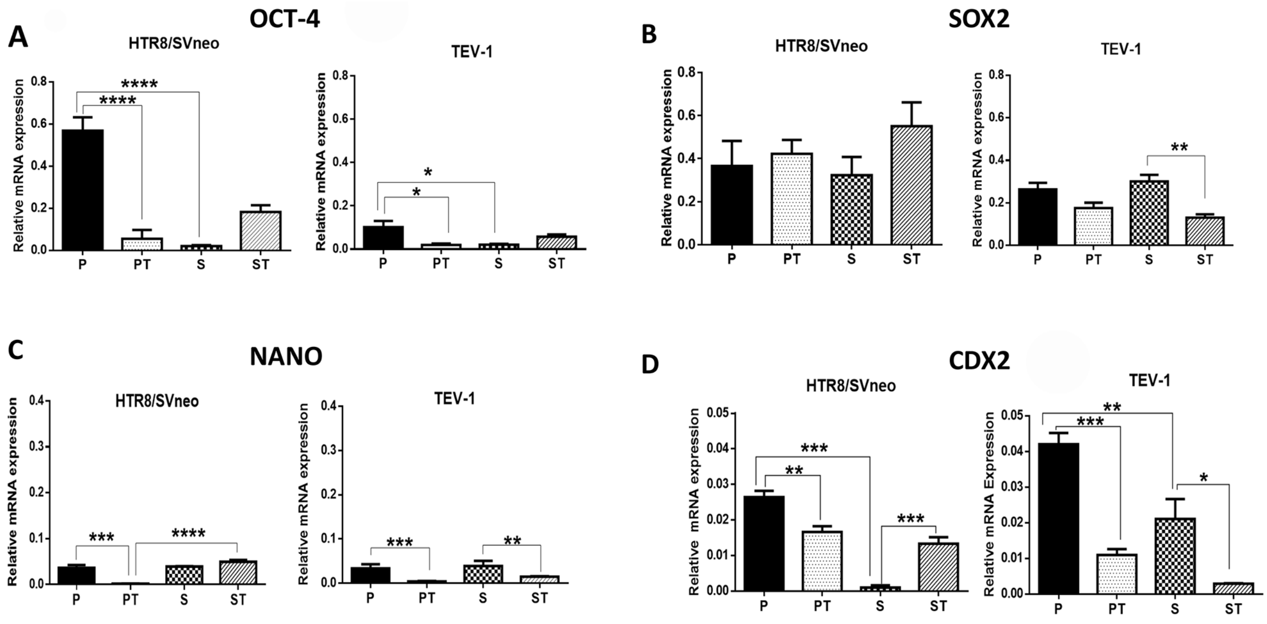

Supplementary Figure 1: Relative mRNA expression of OCT-4, SOX2, NANOG and CDX2 in untreated and treated transformed parental trophoblast cells and spheroids are given in panels **(A) (B) (C)** and **(D)**. Statistical significance was determined using a one-way ANOVA followed by Tukey's test for multiple comparisons. Data represent the mean±SEM of three individual experiments, each performed in triplicate (****p<0.0001; ***p<0.001; **p<0.01; *p<0.05).

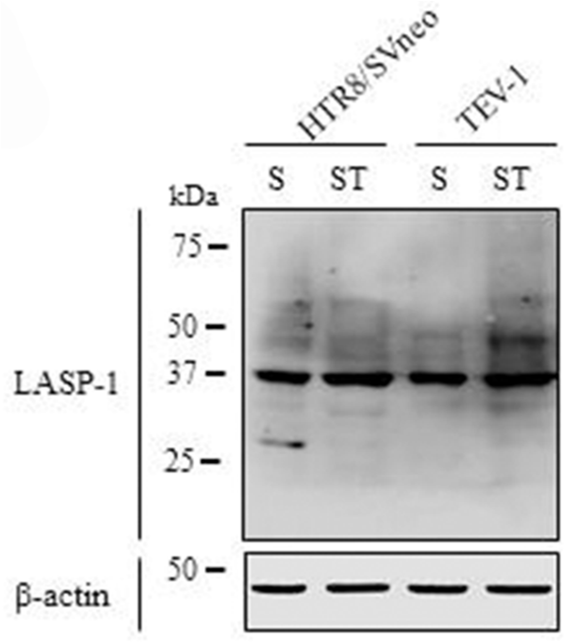

Supplementary Figure 2: LASP1 expression in untreated and treated transformed spheroids. Immunoblots showing the expression of LASP1 and corresponding β-actin loading controls in HTR8/SVneo and TEV-1 untreated and treated transformed spheroids. (confirmation of mass-spectrometry data).
