# Supplementary material for: Identification and characterisation of NANOG+/ OCT-4high/SOX2+ doxorubicin-resistant stem-like cells from transformed trophoblastic cell lines
Source: Oncotarget. 2018 Jan 11;9(6):7054–65. doi: 10.18632/oncotarget.24151 (PMC5805535; doi:10.18632/oncotarget.24151)
Supplement: Supplementary file 2 [file oncotarget-09-7054-s002.pdf]

Table 1: HTR8/SVneo Spheres untreated vs treated up-regulated pathways

Enrichment by Pathway Maps

#	Maps	Total	pValue	Min FDR	HTR8/SVneo_UP p-value	FDR	In Data	Network Objects from Active Data
1	Transport_The role of AVP in regulation of Aquaporin 2 and renal wate	50	3.748E-04	6.372E-03	3.748E-04	6.372E-03	2	MyHC, MYH9
2	Cytoskeleton remodeling_Regulation of actin cytoskeleton by Rho GT	23	1.395E-02	4.918E-02	1.395E-02	4.918E-02	1	MyHC
3	Cell cycle_Initiation of mitosis	26	1.576E-02	4.918E-02	1.576E-02	4.918E-02	1	Lamin B
4	Cytoskeleton remodeling_Thyroliberin in cytoskeleton remodeling	33	1.996E-02	4.918E-02	1.996E-02	4.918E-02	1	Keratin 8
5	Apoptosis and survival_Caspase cascade	34	2.056E-02	4.918E-02	2.056E-02	4.918E-02	1	Lamin B
6	Cytoskeleton remodeling_Keratin filaments	36	2.176E-02	4.918E-02	2.176E-02	4.918E-02	1	Keratin 8
7	Development_MAG-dependent inhibition of neurite outgrowth	37	2.236E-02	4.918E-02	2.236E-02	4.918E-02	1	MyHC
8	Apoptosis and survival_FAS signaling cascades	44	2.654E-02	4.918E-02	2.654E-02	4.918E-02	1	Lamin B
9	wtCFTR and deltaF508 traffic / Membrane expression (normal and CF	47	2.833E-02	4.918E-02	2.833E-02	4.918E-02	1	Keratin 8
10	Cell adhesion_Integrin-mediated cell adhesion and migration	48	2.893E-02	4.918E-02	2.893E-02	4.918E-02	1	MyHC
11	Airway smooth muscle contraction in asthma	56	3.368E-02	5.205E-02	3.368E-02	5.205E-02	1	MyHC
12	N-Glycan biosynthesis p1	72	4.313E-02	5.593E-02	4.313E-02	5.593E-02	1	RIB1
13	Blood coagulation_Platelet microparticle generation	72	4.313E-02	5.593E-02	4.313E-02	5.593E-02	1	MyHC
14	Immune response _CCR3 signaling in eosinophils	77	4.606E-02	5.593E-02	4.606E-02	5.593E-02	1	MyHC
15	Muscle contraction_GPCRs in the regulation of smooth muscle tone	83	4.958E-02	5.619E-02	4.958E-02	5.619E-02	1	MyHC
16	Cytoskeleton remodeling_Cytoskeleton remodeling	102	6.063E-02	6.121E-02	6.063E-02	6.121E-02	1	MyHC
17	Oxidative phosphorylation	103	6.121E-02	6.121E-02	6.121E-02	6.121E-02	1	COX Va
