# Supplementary material for: Identification and characterisation of NANOG+/ OCT-4high/SOX2+ doxorubicin-resistant stem-like cells from transformed trophoblastic cell lines
Source: Oncotarget. 2018 Jan 11;9(6):7054–65. doi: 10.18632/oncotarget.24151 (PMC5805535; doi:10.18632/oncotarget.24151)
Supplement: Supplementary file 3 [file oncotarget-09-7054-s003.pdf]

**Table 2: HTR8/SVneo Spheres untreated vs treated down-regulated pathways**

Enrichment by Pathway Maps

| | | | | | HTR8/SVneo DOWN | | | |
|---|---|---|---|---|---|---|---|---|
| # | Maps | Total | pValue | Min FDR | p-value | FDR | In Data | Network Objects from Active Data |
| 1 | Transport_RAN regulation pathway | 18 | 5.479E-03 | 1.494E-02 | 5.479E-03 | 1.494E-02 | 1 | Ran |
| 2 | Cell cycle_Role of Nek in cell cycle regulation | 32 | 9.727E-03 | 1.494E-02 | 9.727E-03 | 1.494E-02 | 1 | Ran |
| 3 | Cell cycle_Spindle assembly and chromosome separation | 33 | 1.003E-02 | 1.494E-02 | 1.003E-02 | 1.494E-02 | 1 | Ran |
| 4 | Apoptosis and survival_APRIL and BAFF signaling | 39 | 1.185E-02 | 1.494E-02 | 1.185E-02 | 1.494E-02 | 1 | Cyclophilin B |
| 5 | Transcription_Role of Akt in hypoxia induced HIF1 activation | 41 | 1.245E-02 | 1.494E-02 | 1.245E-02 | 1.494E-02 | 1 | PGK1 |
| 6 | Glycolysis and gluconeogenesis | 87 | 2.630E-02 | 2.630E-02 | 2.630E-02 | 2.630E-02 | 1 | PGK1 |
