# Supplementary material for: Identification and characterisation of NANOG+/ OCT-4high/SOX2+ doxorubicin-resistant stem-like cells from transformed trophoblastic cell lines
Source: Oncotarget. 2018 Jan 11;9(6):7054–65. doi: 10.18632/oncotarget.24151 (PMC5805535; doi:10.18632/oncotarget.24151)
Supplement: Supplementary file 4 [file oncotarget-09-7054-s004.pdf]

Table 3: TEV-1 Spheres untreated vs treated up-regulated pathways

Enrichment by Pathway Maps

					TEV-1 UP			
#	Maps	Total	pValue	Min FDR	p-value	FDR	In Data	Network Objects from Active Data
1	Cytoskeleton remodeling_Role of PKA in cytoskeleton reorganisation	40	4.066E-03	4.066E-03	4.066E-03	4.066E-03	1	LASP1
