# Supplementary material for: Identification and characterisation of NANOG+/ OCT-4high/SOX2+ doxorubicin-resistant stem-like cells from transformed trophoblastic cell lines
Source: Oncotarget. 2018 Jan 11;9(6):7054–65. doi: 10.18632/oncotarget.24151 (PMC5805535; doi:10.18632/oncotarget.24151)
Supplement: Supplementary file 5 [file oncotarget-09-7054-s005.pdf]

Table 4: TEV-1 Spheres untreated vs treated down-regulated pathways

Enrichment by Pathway Maps

					TEV-1 DOWN			
#	Maps	Total	pValue	Min FDR	p-value	FDR	In Data	Network Objects from Active Data
1	Immune response_Antigen presentation by MHC class I, classical pathway	54	1.551E-06	3.723E-05	1.551E-06	3.723E-05	3	Calreticulin, PDIA3, [illegible]
2	Immune response_Antigen presentation by MHC class I: cross-presentation	99	9.829E-04	1.179E-02	9.829E-04	1.179E-02	2	Calreticulin, HSP70
3	Proteolysis_Putative ubiquitin pathway	23	1.164E-02	3.896E-02	1.164E-02	3.896E-02	1	HSP70
4	CFTR folding and maturation (normal and CF)	24	1.214E-02	3.896E-02	1.214E-02	3.896E-02	1	HSP70
5	Proteolysis_Role of Parkin in the Ubiquitin-Proteasomal Pathway	24	1.214E-02	3.896E-02	1.214E-02	3.896E-02	1	HSP70
6	Development_Glucocorticoid receptor signaling	25	1.264E-02	3.896E-02	1.264E-02	3.896E-02	1	HSP70
7	Apoptosis and survival_Role of IAP-proteins in apoptosis	31	1.566E-02	3.896E-02	1.566E-02	3.896E-02	1	HSP70
8	Oxidative stress_Role of ASK1 under oxidative stress	34	1.716E-02	3.896E-02	1.716E-02	3.896E-02	1	HSP70
9	Immune response_TLR ligands	34	1.716E-02	3.896E-02	1.716E-02	3.896E-02	1	HSP70
10	Role of Endothelin-1 in inflammation and vasoconstriction in Sickle cell disease	38	1.917E-02	3.896E-02	1.917E-02	3.896E-02	1	P4HB
11	Regulation of degradation of deltaF508-CFTR in CF	39	1.967E-02	3.896E-02	1.967E-02	3.896E-02	1	HSP70
12	Mechanisms of deltaF508 CFTR activation by S-nitrosoglutathione	47	2.366E-02	3.896E-02	2.366E-02	3.896E-02	1	HSP70
13	wtCFTR and deltaF508 traffic / Membrane expression (normal and CF)	47	2.366E-02	3.896E-02	2.366E-02	3.896E-02	1	Calreticulin
14	Proline metabolism	51	2.566E-02	3.896E-02	2.566E-02	3.896E-02	1	P4HB
15	Immune response_HSP60 and HSP70/ TLR signaling pathway	54	2.715E-02	3.896E-02	2.715E-02	3.896E-02	1	HSP70
16	Apoptosis and survival_Endoplasmic reticulum stress response pathway	56	2.814E-02	3.896E-02	2.814E-02	3.896E-02	1	GRP78
17	Transcription_Role of VDR in regulation of genes involved in osteoporosis	61	3.063E-02	3.896E-02	3.063E-02	3.896E-02	1	Calreticulin
18	Neuroprotective action of lithium	63	3.162E-02	3.896E-02	3.162E-02	3.896E-02	1	HSP70
19	Signal transduction_mTORC2 upstream signaling	65	3.261E-02	3.896E-02	3.261E-02	3.896E-02	1	HSP70
20	Transcription_Negative regulation of HIF1A function	66	3.310E-02	3.896E-02	3.310E-02	3.896E-02	1	HSP70
21	Immune response_Sublytic effects of membrane attack complex	68	3.409E-02	3.896E-02	3.409E-02	3.896E-02	1	GRP78
22	Signal transduction_mTORC1 upstream signaling	74	3.706E-02	4.042E-02	3.706E-02	4.042E-02	1	PDIA3
23	SLE genetic marker-specific pathways in antigen-presenting cells (APC)	84	4.198E-02	4.380E-02	4.198E-02	4.380E-02	1	GRP78
24	SLE genetic marker-specific pathways in B cells	100	4.981E-02	4.981E-02	4.981E-02	4.981E-02	1	GRP78
